# Volumetric analysis of the hypothalamic subunits in obstructive sleep apnea

**DOI:** 10.1002/brb3.70026

**Published:** 2024-09-05

**Authors:** Mahdi Mohammadi, Mohammad Ali Oghabian, Sadegh Ghaderi, Maryam Jalali, Shahram Samadi

**Affiliations:** ^1^ Department of Medical Physics and Biomedical Engineering, School of Medicine Tehran University of Medical Sciences Tehran Iran; ^2^ Neuroimaging and Analysis Group, Research Center for Molecular and Cellular Imaging, Advanced Medical Technologies and Equipment Institute Tehran University of Medical Sciences Tehran Iran; ^3^ Department of Neuroscience and Addiction Studies, School of Advanced Technologies in Medicine Tehran University of Medical Sciences Tehran Iran; ^4^ Sleep Breathing Disorders Research Center, Imam Khomeini Hospital Complex, School of Medicine Tehran University of Medical Sciences Tehran Iran; ^5^ Anesthesia, Critical Care and Pain Management Research Center Tehran University of Medical Sciences Tehran Iran

**Keywords:** hypothalamus, MRI, obstructive sleep apnea, polysomnography, volumetry

## Abstract

**Background:**

Obstructive sleep apnea (OSA) is a prevalent sleep disorder that is associated with structural brain damage and cognitive impairment. The hypothalamus plays a crucial role in regulating sleep and wakefulness. We aimed to evaluate hypothalamic subunit volumes in patients with OSA.

**Methods:**

We enrolled 30 participants (15 patients with OSA and 15 healthy controls (HC)). Patients with OSA underwent complete overnight polysomnography (PSG) examination. All the participants underwent MRI. The hypothalamic subunit volumes were calculated using a segmentation technique that trained a 3D convolutional neural network.

**Results:**

Although hypothalamus subunit volumes were comparable between the HC and OSA groups (lowest *p* = .395), significant negative correlations were found in OSA patients between BMI and whole left hypothalamus volume (*R* = –0.654, *p* = .008), as well as between BMI and left posterior volume (*R* = –0.556, *p* = .032). Furthermore, significant positive correlations were found between ESS and right anterior inferior volume (*R* = 0.548, *p* = .042), minimum SpO_2_ and the whole left hypothalamus (*R* = 0.551, *p* = .033), left tubular inferior volumes (*R* = 0.596, *p* = .019), and between the percentage of REM stage and left anterior inferior volume (*R* = 0.584, *p* = .022).

**Conclusions:**

While there were no notable differences in the hypothalamic subunit volumes between the OSA and HC groups, several important correlations were identified in the OSA group. These relationships suggest that factors related to sleep apnea severity could affect hypothalamic structure in patients.

## Graphical abstract

After hypothalamic subunits segmentation, several important correlations were identified in the OSA patients. These correlations suggest that factors related to sleep apnea severity could affect hypothalamic structure in patients.

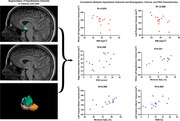

## 1 INTRODUCTION

Obstructive sleep apnea (OSA) is a sleep disorder characterized by partial or total obstruction of the upper airway during sleep, resulting in lower oxygen levels and alterations in blood pressure alterations (Dempsey et al., 2010; Najafi et al., 2021; Park & Kim, 2023; Tummala et al., 2016). It is linked to structural brain damage, abnormalities in various functions, and cognitive issues (Tummala et al., 2016; Zhang et al., 2013). OSA significantly affects people's daily lives and work performance and is associated with long‐term health concerns such as cardiovascular disease and psychological disorders (Drager et al., 2013; Olaithe et al., 2018; Wheaton et al., 2012). Sleep fragmentation, poor sleep efficiency, and intermittent hypoxia all contribute to cognitive impairments in those with OSA (Daurat et al., 2008; Olaithe et al., 2018; Verstraeten, 2007).

The hypothalamus, a small but vital structure in the brain, is responsible for a variety of homeostatic activities that are essential for survival (Adamantidis & de Lecea, 2023). It controls thermoregulation, metabolism, physiological fluid balance, reproductive and aggressive behaviors, and sleep/wakefulness (Adamantidis & de Lecea, 2023; Mickelsen et al., 2019; Moffitt et al., 2018). The hypothalamus, which serves as a hub for central and peripheral signals, regulates sleep/wakefulness and the endocrine system (Gnoni et al., 2023; Hiller & Ishii, 2018; Morton et al., 2014). It serves as a “flip‐flop switch, ” which may promote sleep while inhibiting awakening and vice versa, giving it control over various states. During awake and rapid eye movement (REM) sleep, the hypothalamus stimulates pathways that activate the cerebral cortex. When the body prepares for sleep, the hypothalamus can efficiently turn pathways that cause wakefulness and activate the pathways that promote sleep. This involves blocking signals in the brainstem and hypothalamus, which can trigger arousal and prevent falling asleep (Saper et al., 2010; Saper et al., 2005; Vgontzas & Pavlović, 2018).

Magnetic resonance imaging (MRI) is a highly valuable diagnostic technique for studying brain structures and areas (Ghaderi et al., 2023; Yousaf et al., 2018). Currently, it is possible to calculate the volume of any brain structure or region with excellent accuracy and robustness using deep‐learning‐based analytical tools for automated segmentation (Billot et al., 2020; Greve et al., 2021; Park & Kim, 2023). Assessing volume changes in subcortical regions using this approach may substantially aid in diagnosing the effects of diseases or their potential origins.

Structural changes in the brains of patients with OSA have been the subject of several studies (Kim et al., 2016; Macey et al., 2018; Ramos et al., 2021). These studies have focused on changes in the volume or thickness of various locations; however, there have been no investigations of hypothalamic subunit volumes, which are essential players in the sleep/wake cycle (Park & Kim, 2023).

In this study, we evaluated hypothalamic subunit volumes in patients with OSA as well as the correlation between demographic or clinical factors and brain volumes.

## 2 METHODS

### 2.1 Ethical statement

All participants provided written informed consent, and the study protocol and design were approved by Tehran University of Medical Sciences (IR.TUMS.MEDICINE.REC.1400.1069).

### 2.2 Participants

We examined patients who were newly diagnosed with obstructive sleep apnea (OSA) and had not received any prior treatment. These patients were identified at the sleep disorder clinic of the Imam Khomeini Hospital Complex. The inclusion criteria for OSA were as follows: (1) confirmation of OSA by a sleep disorders specialist based on a comprehensive sleep evaluation including PSG results, questionnaires, interviews and clinical examinations (the most important inclusion criteria were: STOP‐BANG questionnaire score of 3 or more, apnea–hypopnea index (AHI) greater than 5, accompanying symptoms during sleep, such as maintenance insomnia and snoring, morning symptoms, such as unrefreshing sleep, dry throat, and headaches); (2) absence of any other medical (diabetes, coronary heart, liver and kidney, endocrine, and metabolic diseases) or neurological disorders besides OSA, as confirmed by the patient's medical history and evaluation by relevant medical specialists; (3) no evidence of structural brain lesions upon visual inspection of brain MRI scans; (4) normal cognitive function, as assessed by the Montreal Cognitive Assessment (MoCA) test administered by a neuropsychologist; and (5) no history of alcohol or illicit drug abuse or current use of psychoactive medications. All patients underwent comprehensive clinical interviews. To assess the healthy sleep status of the control group, a sleep disorder specialist evaluated and administered the STOP‐BANG questionnaire, as part of the diagnostic criteria. The Epworth Sleepiness Scale (ESS) was used as a subjective measure of all participants' sleepiness. Finally, 15 patients with OSA and 15 healthy controls were enrolled in this study. All the enrolled participants underwent MRI using a specific protocol.

### 2.3 Overnight polysomnography

Patients with OSA underwent complete overnight PSG examination. PSG recordings were conducted using a *miniScreen Pro device (Lowenstein Medical®, Bad Ems, Germany)* and PSG data were analyzed using *miniScreenpro software (Lowenstein Medical®, Bad Ems, Germany)*. All PSG‐reporting parameters were carefully reviewed and confirmed by an experienced specialist in sleep disorders.

Standard polysomnography (PSG) procedure was performed under the same conditions for all patients. The key components of the standard PSG testing included the use of all electroencephalogram (EEG) derivations, two electrooculograms (EOGs), three electromyograms (EMGs) for chin movement (one in the medial position and two in bilateral positions), two EMGs for leg movement (one for each leg), two electrocardiogram (ECG) leads on the chest, body position sensors, nasal flow monitoring, a small microphone for snoring monitoring, abdominal and thoracic belts to assess the respiratory effort and differentiate OSA from central sleep apnea (CSA), and pulse oximetry to measure blood oxygen saturation.

This study utilized several important PSG output parameters, including AHI, oxygen desaturation index (ODI), snoring index (SI), arousal index (AI), rapid eye movement (REM) latency, minimum and mean levels of oxygen saturation (SpO_2_), and the average percentage of each sleep stage (N1%, N2%, N3%, and REM%) in total sleep time (TST).

### 2.4 MRI acquisition

All participants underwent three‐dimensional T1‐weighted magnetization prepared rapid gradient echo (MPRAGE) MRI using a 3.0T MR scanner (MAGNETOM Prisma; Siemens Healthcare, Germany) equipped with a 64‐channel head/neck coil at the National Brain Mapping Laboratory (NBML). The MRI acquisition parameters were as follows: TI = 900 ms, TR/TE = 1840/2.43 ms, flip angle = 8°, and isotropic voxel size = 1 mm^3^. Standard brain MRI protocols, including FLAIR and T2‐weighted imaging, were used to ensure the absence of structural lesions.

### 2.5 MRI data processing

Axial T1‐w volumetric images were analyzed using the FreeSurfer version 7.4.1 software (http://surfer.nmr.mgh.harvard.edu). Volumetric and surface‐based segmentation were performed by FreeSurfer using a template‐driven methodology.

Motion correction, skull stripping, intensity normalization, Talairach transformation, volumetric registration, subcortical structure segmentation, white matter (WM) and gray matter (GM) segmentation, tessellation, smoothing, inflating, spherical mapping and registration, and cortical mapping and parcellation are the steps involved in image processing.

First, our three‐dimensional T1‐weighted MRI data were processed using the FreeSurfer “recon‐all” command. Although this command provides volume measurements for various brain structures, it does not include the hypothalamus. Second, we utilized the hypothalamic subunit script to segment the hypothalamic subunits, distinguishing between the right and left sides and obtaining their absolute volumes. The specific subunits included anterior inferior, anterior superior, posterior, tubular inferior, and tubular superior (Billot et al., 2020). The nuclei within each hypothalamic subunit are shown in Table 1.

**TABLE 1:** The nuclei within each hypothalamic subunit.

Subunits	Nuclei
Anterior inferior	Suprachiasmatic nucleus; supraoptic nucleus (SON)
Anterior superior	Preoptic area; paraventricular nucleus (PVN)
Posterior	Mamillary body (including medial and lateral mamillary nuclei); lateral hypothalamus; tuberomamillary nucleus (TMN)
Tubular inferior	Infundibular (or arcuate) nucleus; ventromedial nucleus; SON; lateral tubular nucleus; TMN
Tubular superior	Dorsomedial nucleus; PVN; lateral hypothalamus

This segmentation technique uses a 3D convolutional neural network (3D‐CNN). To ensure robustness against variations in acquisition parameters (such as sequence, platform, bias field, and head positioning) and anatomical differences (such as atrophy patterns related to aging or different pathologies), the segmentation model was trained using aggressive data‐augmentation techniques. Before inclusion in the group analysis, all segmentations were visually inspected for accuracy in order to address potential errors in the automated procedure. The expert visual quality control confirmed the reliability of our segmentation. An independent reviewer, blinded to subject characteristics, such as age, sex, and condition, found that this method produced consistent results. This validation demonstrated the robustness of the proposed approach (Billot et al., 2020).

### 2.6 Statistical analysis

Continuous variables are reported as mean (SD), while categorical variables are presented as n counts (%). Prior to analysis, the Kolmogorov–Smirnov test was used to assess the normality of data distribution. The independent samples *t*‐test or Mann–Whitney *U* test was used to compare continuous variables, whereas the *χ* ^2^ or Fisher exact tests were used for categorical variables. Correlation analysis was performed and the results were reported as Pearson's or Spearman's correlation coefficients. All tests were two‐tailed, with a significance level of 0.05. Corrections for multiple testing were performed in the statistical analyses. Bonferroni correction was used to account for the increased risk of type 2 errors when conducting multiple comparisons. SPSS Statistics software (IBM Corporation) was used for all the analyses.

## 3 RESULTS

### 3.1 Demographic, clinical, and PSG characteristics

Our study included 15 OSA patients and 15 age‐matched healthy controls (HC). Table 2 shows the participants' demographic and clinical characteristics, and Table 3 shows the PSG characteristics of patients with OSA.

**TABLE 2:** Demographic and clinical characteristics of the participants.

	OSA (*N* = 15)	HC (*N* = 15)	*p*‐Value^*^	Statistical test
Sex, male (%)	12 (80%)	12 (80%)	1.00	Chi‐squared
Age (years)	47.67 ± 10.16	47.80 ± 10.51	.97	Independent *t*‐test
BMI (kg/m^2^)	29.63 ± 3.05	27.98 ± 3.12	.15	Independent *t*‐test
Right‐handedness	15 (100%)	15 (100%)	1.00	Chi‐squared
Disease duration (month)	36.07 ± 22.09	NA	NA	NA
STOP‐BANG (score)	5.22 ± 2.46	0.83 ± 0.71	3.326 × 10^−7^	Mann–Whitney *U*
ESS (score)	12.14 ± 6.11	4.06 ± 2.18	.00004	Independent *t*‐test

*p < .05 was considered significant.

*Note*: Data presented as mean ± SD for continuous variables or *n* counts (%) for categorical variables.

OSA, obstructive sleep apnea; HC: healthy control; BMI, body mass index; ESS, Epworth Sleepiness Scale; NA, not applicable; AHI, apnea–hypopnea index; ODI, oxygen desaturation index; SI, snoring index; AI, arousal index; REM, rapid eye movement; SpO_2_, oxygen saturation.

**TABLE 3:** PSG characteristics of the OSA patients.

AHI (number/h)	57.96 ± 38.17
ODI (number/h)	49.02** ±** 28.87
SI (number/h)	304.81** ±** 217.29
AI (number/h)	35.73** ±** 32.65
REM Latency (min)	158.41** ±** 99.86
Minimum SpO_2_ (%)	74.80** ±** 9.02
Mean SpO_2_ (%)	91.53** ±** 1.95
N1 (%)	25.81** ±** 20.85
N2 (%)	55.72** ±** 16.90
N3 (%)	9.08** ±** 7.61
REM (%)	9.45** ±** 5.45

*Note*: Data presented as mean ± SD.

OSA, obstructive sleep apnea; AHI, apnea–hypopnea index; ODI, oxygen desaturation index; SI, snoring index; AI, arousal index; REM, rapid eye movement; SpO_2_, oxygen saturation.

### 3.2 Hypothalamic subunit volumes in OSA and HC

**FIGURE 1:**
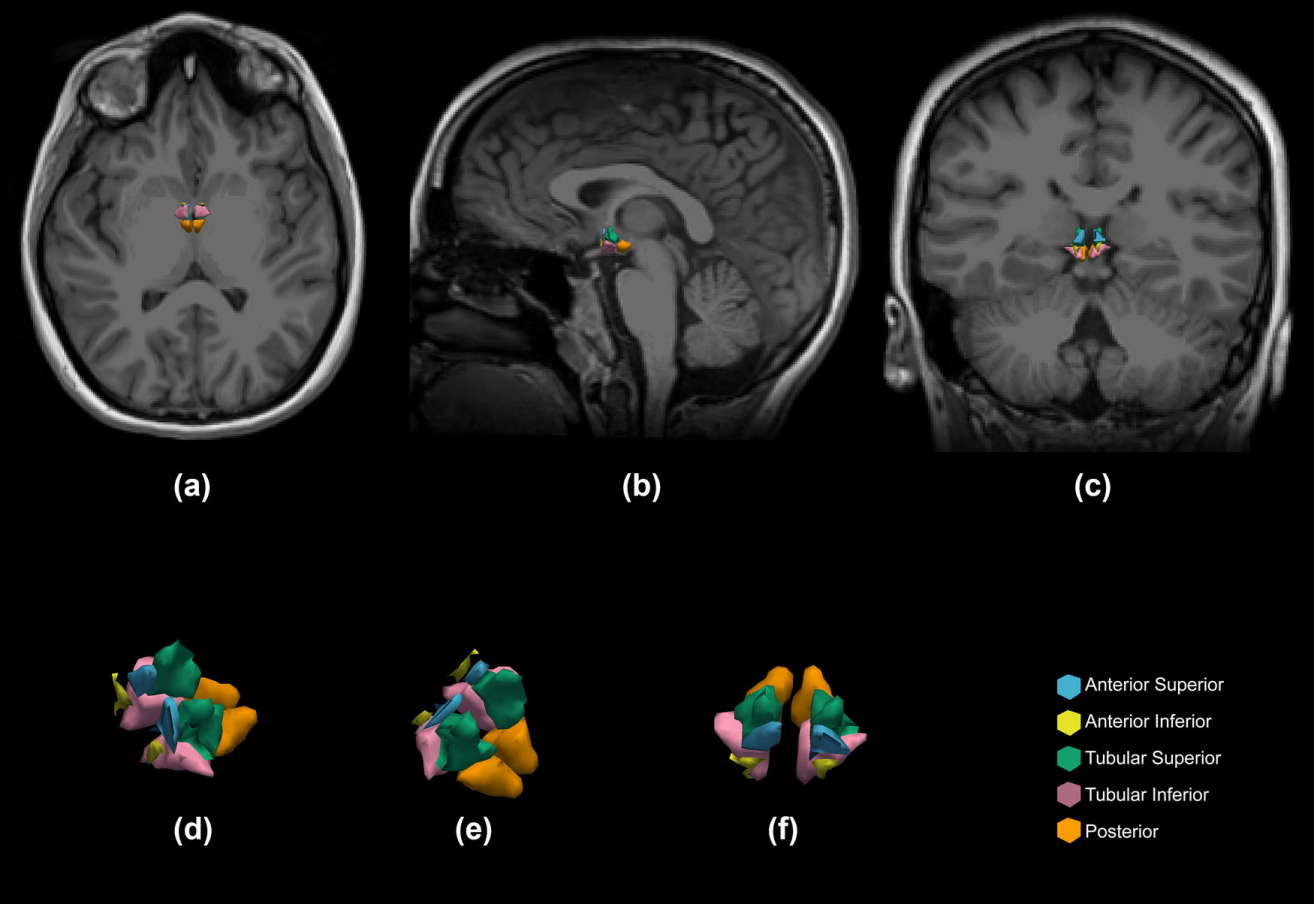
Example of graphical representation of segmentation output of hypothalamic subunits: (a) axial view, (b) sagittal view, (c) coronal view, and (d), (e), and (f) 3D views.

The segmentation output of one of the hypothalamic subunits of the participants is shown in Figure 1. As shown in Table 2, there were no significant differences between the two groups in terms of age, sex, and BMI. However, in the analysis and comparison of hypothalamic volumes, the effect of these items along with intracranial volume (ICV) was corrected so that the results could be analyzed more reliably. There were no significant differences in hypothalamic subunit volumes between the OSA and HC groups. Table 4 presents the results of the volumetric analysis.

**TABLE 4:** Hypothalamic subunit volumes in OSA and HC.

	OSA (*N* = 15)	HC (*N* = 15)	*p*‐Value^*^	Statistical test
Whole hypothalamus	867.85 ± 54.50	872.40 ± 79.81	.395	Mann–Whitney *U*
Whole right hypothalamus	426.51 ± 26.59	433.23 ± 36.84	.571	Independent *t*‐test
Whole left hypothalamus	441.33 ± 31.46	439.16 ± 47.17	.885	Mann–Whitney *U*
Right anterior inferior	19.42 ± 3.56	19.48 ± 4.54	.971	Independent *t*‐test
Right anterior superior	24.73 ± 3.74	24.21 ± 4.88	.744	Independent *t*‐test
Right posterior	125.80 ± 13.17	127.06 ± 16.49	.819	Independent *t*‐test
Right tubular inferior	136.39 ± 11.56	140.06 ± 13.57	.432	Independent *t*‐test
Right tubular superior	120.15 ± 10.59	122.41 ± 13.80	.443	Mann–Whitney *U*
Left anterior inferior	19.30 ± 3.24	18.90 ± 3.94	.764	Independent *t*‐test
Left anterior superior	26.11 ± 3.32	26.71 ± 3.30	.623	Independent *t*‐test
Left posterior	127.51 ± 17.51	126.34 ± 14.24	.842	Independent *t*‐test
Left tubular inferior	152.32 ± 14.21	147.69 ± 22.32	.503	Independent *t*‐test
Left tubular superior	116.08 ± 10.35	119.50 ± 16.47	.502	Independent *t*‐test

*p < .05 was considered significant.

*Note*: All volumes reported as mean ± SD in mm^3^.

### 3.3 The correlation between clinical and PSG characteristics and hypothalamic subunit volumes

**FIGURE 2:**
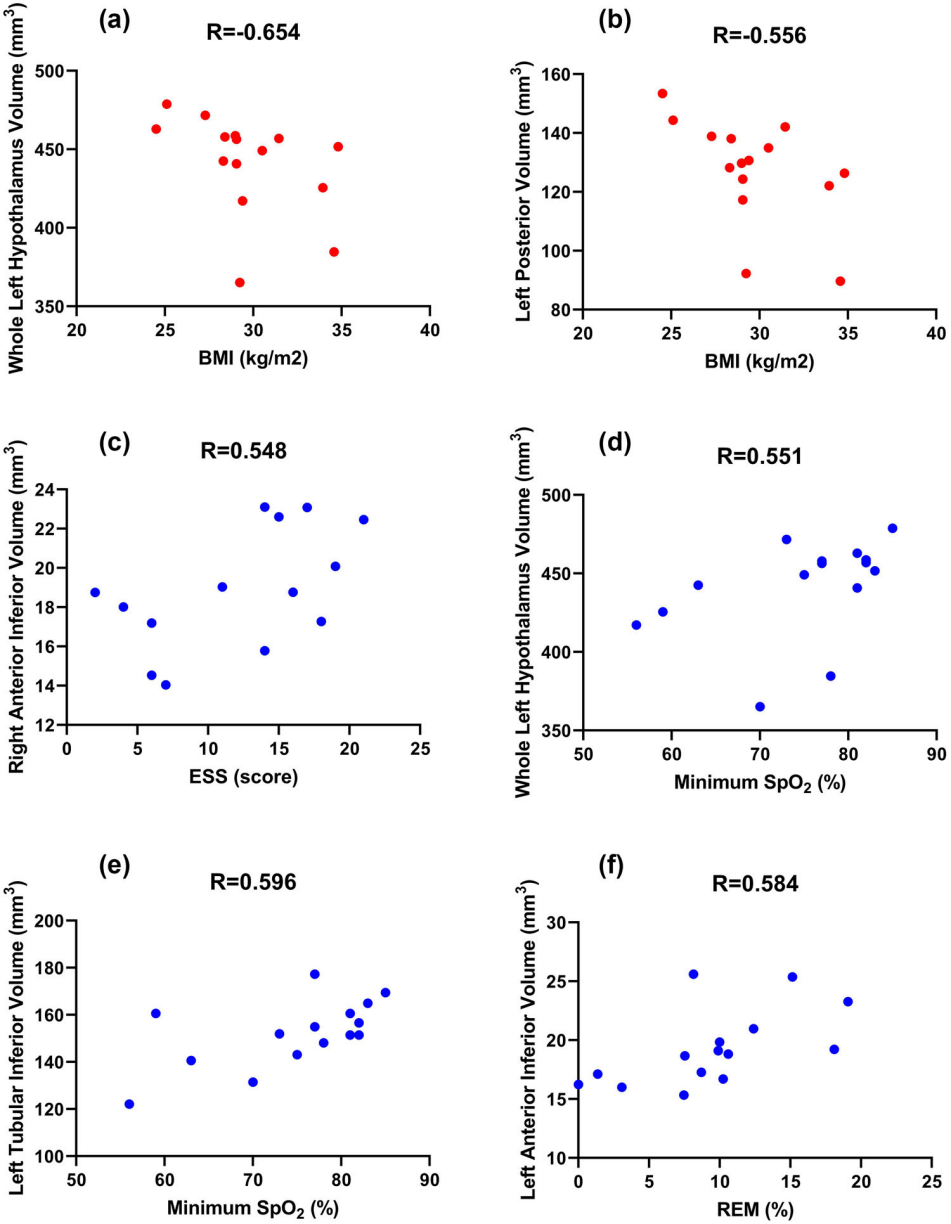
Correlation graphs for variables that were significant in OSA but not in HC: (a) BMI and whole left hypothalamus volume; (b) BMI and left posterior volume; (c) ESS and right anterior inferior volume; (d) Minimum SpO_2_ and whole left hypothalamus volume; (e) Minimum SpO_2_ and left tubular inferior volume; and (f) REM and left anterior inferior volume.

The correlation between clinical and PSG characteristics and the hypothalamic subunit volume was examined. Significant negative correlations were found between BMI and whole left hypothalamus volume as well as between BMI and left posterior volume. Furthermore, significant positive correlations were found between ESS and right anterior inferior volume, between minimum SpO_2_ and the whole left hypothalamus and left tubular inferior volumes, and between the percentage of REM stage and left anterior inferior volume. Table 5 and Figure 2 present the correlations that were significant in the OSA group, but not in the HC group. All the correlation analyses are reported in Tables S1–S3.

**TABLE 5:** Significant correlations in OSA.

	Correlation coefficient (*R*)	*p*‐Value^*^	Correlation method
ESS and right anterior inferior volume	0.548	.042	Pearson
Minimum SpO_2_ and whole left hypothalamus volume	0.551	.033	Spearman
Minimum SpO_2_ and left tubular inferior volume	0.596	.019	Pearson
REM and left anterior inferior volume	0.584	.022	Pearson

*p < .05 was considered significant.

## 4 DISCUSSION

In this study, we utilized a precise and robust 3D‐CNN segmentation tool to assess the hypothalamic subunit volumes in patients with OSA. Notably, no comparable studies benchmark our results, making our findings a potential reference point for future studies.

Although hypothalamus subunit volumes were comparable between the HC and OSA groups, the OSA group displayed multiple significant correlations. This difference in correlational patterns between the groups indicated that the hypothalamus may be organized differently in patients with OSA.

Our findings showed a negative correlation between BMI and both left posterior and whole left hypothalamic volume. It has been proposed that intermittent hypoxemia from OSA could elevate oxidative stress and inflammation levels in the hypothalamus, especially when combined with metabolic dysregulation from excess adiposity (Biglari et al., 2021; Bonsignore et al., 2012; Jehan et al., 2017; Romero‐Corral et al., 2010; Wolk et al., 2003). This poses particular risks, as neuronal populations, such as POMC/NPY neurons, appear inherently vulnerable. Chronic intermittent hypoxia accelerates the pruning/reshaping of less resilient regions, such as the left posterior hypothalamus, potentially hastened by the interaction between hypoxia and elevated BMI (Biglari et al., 2021; Marraudino et al., 2021; Paeger et al., 2017; Sohn, 2015). These processes cause a reduction in the hypothalamic subunit volume. The fact that all study patients were right‐handed could be the reason for the observed correlation, particularly in the left hemisphere. Some studies have shown a positive correlation between BMI and hypothalamus volume and have hypothesized that an obese person's high‐fat diet is the primary cause of hypothalamic inflammation, whereas others have shown no correlation between them (Brown et al., 2023; Thomas et al., 2019a; Thomas et al., 2019b).

Positive correlations were found between ESS scores and right anterior inferior volume, suggesting that a decrease in the volume of this region underlies increased daily sleepiness in patients with OSA. Given that the suprachiasmatic nucleus (SCN) is situated in the anterior inferior region of the hypothalamus, its decrease could cause greater sleepiness in patients with OSA, as determined using the ESS questionnaire. The SCN controls circadian rhythms by regulating melatonin production in the pineal gland, which affects sleep induction via signals from photosensitive ganglion cells (Hastings et al., 2018; Varadarajan et al., 2018).

The minimum nocturnal SpO_2_ in patients positively correlated with the left hypothalamus and left tubular inferior volumes, indicating higher sensitivity to hypoxic stress levels. The core circuits of the infundibular, supraoptic, and tuberomamillary nuclei, which play an important role in physiological responses to hypoxia, explain their heightened vulnerability in terms of structure and function during periods of intermittent hypoxia (Gerlach et al., 2021; King et al., 2012; King et al., 2013).

A positive correlation was found between the percentage of the REM stage and volume of the left anterior inferior region, suggesting that OSA severity could increase during this stage. Owing to the recruitment of more inhibitory inputs to cranial motor pools during REM sleep, airway closure could worsen in patients with OSA (Fraigne et al., 2014; Peregrim et al., 2013). However, during REM sleep, the anterior hypothalamus has a less significant role in controlling respiration in response to temperature (Harding et al., 2020; Harper et al., 2014). When breathing becomes less thermally driven during REM, an increase in volume can counteract any potential risk resulting from decreased function.

The non‐significance of these subunit changes in OSA patients compared to healthy controls in this study can serve as a foundation for future studies, as no study has specifically studied the volume of the hypothalamic subunits in OSA patients. However, our findings regarding the total volume of the hypothalamus are in contrast to previous studies that have indicated a significant reduction in the volume of the hypothalamus in these patients (Deyang et al., 2023; Filipovic et al., 2021) Given that we used a highly accurate and robust 3D‐CNN model, this might be one reason for the lack of a significant volume difference (Billot et al., 2020).

This was the primary study using a highly accurate and robust 3D‐CNN segmentation tool to evaluate the hypothalamic subunit volumes in patients with OSA. However, this study had several limitations. First, the study had a small sample size and may include some OSA patients with a short disease duration, which limits the generalizability of the findings. Second, owing to the retrospective nature of the study and comparison between patients with OSA and healthy controls, it was not possible to establish a temporal association. Therefore, it remains uncertain whether the altered hypothalamus volume is the cause or result of OSA. Third, the inclusion of the control group without a PSG study, relying solely on clinical examination and a STOP‐BANG questionnaire, may have included individuals with undetected moderate sleep apnea. In addition, the lack of PSG data of healthy controls and their correlations with hypothalamic subunit volumes can affect the discussion regarding the involvement of hypothalamic subunit volumes in the pathology of OSA. Fourth, the relatively short average disease duration in the subjects of the study (22.09 + 36.07 months) may be associated with the lack of volume change in the hypothalamic subunits, and additional time will probably be required for observation.

## 5 CONCLUSIONS

Based on these findings, although there were no notable variances in hypothalamic subunit volumes between patients in the OSA and HC groups, several important correlations were identified in the OSA groups. Higher age and BMI were associated with smaller volumes of specific hypothalamic subunits. Additionally, worse sleepiness, as measured by ESS, lower minimum SpO_2_ levels, and a lower percentage of REM sleep were correlated with smaller volumes in certain subunits of patients with OSA. These relationships suggest that factors related to sleep apnea severity could affect the hypothalamic structure in patients. Moreover, the differences in correlational patterns between the groups indicated that the hypothalamus could be organized differently in patients with OSA. Further research is warranted to determine whether structural changes in the hypothalamus contribute to the development and progression of OSA, or are a consequence of this condition. Overall, the results of this study provide insights that could aid in determining the underlying pathological mechanisms of OSA in future studies.

## AUTHOR CONTRIBUTIONS

**Mahdi Mohammadi**: Conceptualization; software; data curation; visualization; writing—original draft; writing—review and editing; formal analysis; investigation; validation. **Mohammad Ali Oghabian**: Conceptualization; methodology; supervision; validation; formal analysis; writing—review and editing. **Sadegh Ghaderi**: Conceptualization; methodology; software; validation; formal analysis; writing—review and editing. **Maryam Jalali**: Software; data curation; writing—original draft; investigation; writing—review and editing. **Shahram Samadi**: Conceptualization; methodology; supervision; validation; formal analysis; writing—review and editing.

## CONFLICT OF INTEREST STATEMENT

The authors declare that they have no conflicts of interest.

## FUNDING

This research was conducted without external funding.

### PEER REVIEW

The peer review history for this article is available at https://publons.com/publon/10.1002/brb3.70026.

## Supporting information

Table S1. Correlations between demographic and clinical characteristics and hypothalamic volumes in OSA.
Table S2. Correlations between demographic and clinical characteristics and hypothalamic volumes in healthy controls.
Table S3. Correlations between PSG characteristics and hypothalamic volumes in OSA.

## Data Availability

This article contains all the data produced or analyzed during this investigation. Further inquiries should be forwarded to the corresponding author.
