# Supplementary material for: Volumetric analysis of the hypothalamic subunits in obstructive sleep apnea
Source: Brain Behav. 2024 Sep 5;14(9):e70026. doi: 10.1002/brb3.70026 (PMC11376441; doi:10.1002/brb3.70026)
Supplement: Supplementary file 1 — Table S1. Correlations between demographic and clinical characteristics and hypothalamic volumes in OSA. Table S2. Correlations between demographic and clinical characteristics and hypothalamic volumes in healthy controls. Table S3. Correlations between PSG characteristics and hypothalamic volumes in OSA. [file BRB3-14-e70026-s001.docx]

| Supplementary Table 1. Correlations between demographic and clinical characteristics and hypothalamic volumes in OSA | | | | |
| --- | --- | --- | --- | --- |
| Correlation Between | | **Correlation Coefficient (R)** | **p-value^*^** | **Correlation Method** |
| Gender | Whole Hypothalamus | -0.424 | 0.115 | Spearman |
|  | Whole Right Hypothalamus | -0.347 | 0.205 | Spearman |
|  | Whole Left Hypothalamus | -0.473 | 0.075 | Spearman |
|  | Right Anterior Inferior | -0.039 | 0.891 | Spearman |
|  | Right Anterior Superior | -0.347 | 0.205 | Spearman |
|  | Right Posterior | -0.501 | 0.057 | Spearman |
|  | Right Tubular Inferior | -0.193 | 0.491 | Spearman |
|  | Right Tubular Superior | -0.471 | 0.089 | Spearman |
|  | Left Anterior Inferior | 0.000 | 1 | Spearman |
|  | Left Anterior Superior | -0.329 | 0.231 | Spearman |
|  | Left Posterior | -0.412 | 0.127 | Spearman |
|  | Left Tubular Inferior | -0.193 | 0.491 | Spearman |
|  | Left Tubular Superior | -0.481 | 0.069 | Spearman |
| Age | Whole Hypothalamus | -0.171 | 0.543 | Spearman |
|  | Whole Right Hypothalamus | -0.147 | 0.600 | Pearson |
|  | Whole Left Hypothalamus | -0.205 | 0.464 | Spearman |
|  | Right Anterior Inferior | 0.012 | 0.965 | Pearson |
|  | Right Anterior Superior | -0.364 | 0.182 | Spearman |
|  | Right Posterior | -0.191 | 0.496 | Pearson |
|  | Right Tubular Inferior | 0.129 | 0.646 | Spearman |
|  | Right Tubular Superior | -0.494 | 0.061 | Pearson |
|  | Left Anterior Inferior | 0.012 | 0.965 | Pearson |
|  | Left Anterior Superior | -0.308 | 0.264 | Pearson |
|  | Left Posterior | -0.219 | 0.434 | Pearson |
|  | Left Tubular Inferior | 0.038 | 0.893 | Pearson |
|  | Left Tubular Superior | -0.219 | 0.432 | Pearson |
| BMI | Whole Hypothalamus | -0.440 | 0.101 | Spearman |
|  | Whole Right Hypothalamus | -0.192 | 0.494 | Pearson |
|  | Whole Left Hypothalamus | -0.467 | 0.072 | Spearman |
|  | Right Anterior Inferior | 0.020 | 0.944 | Pearson |
|  | Right Anterior Superior | 0.104 | 0.713 | Spearman |
|  | Right Posterior | -0.287 | 0.299 | Pearson |
|  | Right Tubular Inferior | 0.013 | 0.965 | Spearman |
|  | Right Tubular Superior | -0.388 | 0.153 | Pearson |
|  | Left Anterior Inferior | 0.035 | 0.900 | Pearson |
|  | Left Anterior Superior | -0.434 | 0.106 | Pearson |
|  | Left Posterior | -0.294 | 0.271 | Pearson |
|  | Left Tubular Inferior | 0.027 | 0.923 | Pearson |
|  | Left Tubular Superior | -0.370 | 0.175 | Pearson |
| Disease Duration | Whole Hypothalamus | -0.369 | 0.175 | Spearman |
|  | Whole Right Hypothalamus | -0.258 | 0.354 | Pearson |
|  | Whole Left Hypothalamus | -0.171 | 0.542 | Spearman |
|  | Right Anterior Inferior | -0.415 | 0.124 | Pearson |
|  | Right Anterior Superior | -0.355 | 0.194 | Spearman |
|  | Right Posterior | -0.473 | 0.075 | Pearson |
|  | Right Tubular Inferior | 0.311 | 0.259 | Spearman |
|  | Right Tubular Superior | -0.081 | 0.773 | Pearson |
|  | Left Anterior Inferior | -0.103 | 0.714 | Pearson |
|  | Left Anterior Superior | -0.384 | 0.158 | Pearson |
|  | Left Posterior | -0.313 | 0.256 | Pearson |
|  | Left Tubular Inferior | 0.481 | 0.069 | Pearson |
|  | Left Tubular Superior | -0.420 | 0.119 | Pearson |
| STOP-BANG | Whole Hypothalamus | 0.501 | 0.057 | Spearman |
|  | Whole Right Hypothalamus | 0.427 | 0.117 | Pearson |
|  | Whole Left Hypothalamus | 0.526 | 0.063 | Spearman |
|  | Right Anterior Inferior | 0.342 | 0.273 | Pearson |
|  | Right Anterior Superior | 0.384 | 0.158 | Spearman |
|  | Right Posterior | 0.365 | 0.181 | Pearson |
|  | Right Tubular Inferior | 0.352 | 0.191 | Spearman |
|  | Right Tubular Superior | 0.319 | 0.266 | Pearson |
|  | Left Anterior Inferior | 0.015 | 0.954 | Pearson |
|  | Left Anterior Superior | 0.228 | 0.415 | Pearson |
|  | Left Posterior | 0.526 | 0.063 | Pearson |
|  | Left Tubular Inferior | 0.543 | 0.057 | Pearson |
|  | Left Tubular Superior | 0.374 | 0.168 | Pearson |
| ESS | Whole Hypothalamus | 0.218 | 0.454 | Spearman |
|  | Whole Right Hypothalamus | -0.153 | 0.601 | Pearson |
|  | Whole Left Hypothalamus | 0.218 | 0.454 | Spearman |
|  | Right Anterior Inferior | 0.548 | 0.042 | Pearson |
|  | Right Anterior Superior | 0.247 | 0.395 | Spearman |
|  | Right Posterior | -0.180 | 0.537 | Pearson |
|  | Right Tubular Inferior | -0.062 | 0.834 | Spearman |
|  | Right Tubular Superior | 0.074 | 0.801 | Pearson |
|  | Left Anterior Inferior | -0.144 | 0.624 | Pearson |
|  | Left Anterior Superior | 0.471 | 0.089 | Pearson |
|  | Left Posterior | -0.089 | 0.763 | Pearson |
|  | Left Tubular Inferior | -0.290 | 0.314 | Pearson |
|  | Left Tubular Superior | 0.319 | 0.266 | Pearson |
| *p<0.05 was considered significant | | | | |

| Supplementary Table 2. Correlations between demographic and clinical characteristics and hypothalamic volumes in Healthy Controls | | | | |
| --- | --- | --- | --- | --- |
| Correlation Between | | **Correlation Coefficient (R)** | **p-value^*^** | **Correlation Method** |
| Gender | Whole Hypothalamus | -0.231 | 0.407 | Spearman |
|  | Whole Right Hypothalamus | -0.386 | 0.156 | Spearman |
|  | Whole Left Hypothalamus | -0.193 | 0.491 | Spearman |
|  | Right Anterior Inferior | 0.116 | 0.681 | Spearman |
|  | Right Anterior Superior | -0.193 | 0.154 | Spearman |
|  | Right Posterior | -0.463 | 0.082 | Spearman |
|  | Right Tubular Inferior | -0.270 | 0.330 | Spearman |
|  | Right Tubular Superior | -0.424 | 0.115 | Spearman |
|  | Left Anterior Inferior | 0.386 | 0.156 | Spearman |
|  | Left Anterior Superior | 0.000 | 1 | Spearman |
|  | Left Posterior | -0.463 | 0.082 | Spearman |
|  | Left Tubular Inferior | -0.193 | 0.491 | Spearman |
|  | Left Tubular Superior | -0.116 | 0.681 | Spearman |
| Age | Whole Hypothalamus | -0.497 | 0.100 | Pearson |
|  | Whole Right Hypothalamus | -0.482 | 0.062 | Pearson |
|  | Whole Left Hypothalamus | -0.387 | 0.154 | Pearson |
|  | Right Anterior Inferior | -0.035 | 0.902 | Pearson |
|  | Right Anterior Superior | -0.302 | 0.274 | Pearson |
|  | Right Posterior | -0.365 | 0.181 | Pearson |
|  | Right Tubular Inferior | -0.501 | 0.057 | Pearson |
|  | Right Tubular Superior | -0.543 | 0.057 | Spearman |
|  | Left Anterior Inferior | 0.064 | 0.821 | Pearson |
|  | Left Anterior Superior | -0.288 | 0.297 | Pearson |
|  | Left Posterior | -0.027 | 0.923 | Pearson |
|  | Left Tubular Inferior | -0.228 | 0.415 | Pearson |
|  | Left Tubular Superior | -0.492 | 0.064 | Pearson |
| BMI | Whole Hypothalamus | 0.170 | 0.544 | Pearson |
|  | Whole Right Hypothalamus | 0.071 | 0.802 | Pearson |
|  | Whole Left Hypothalamus | 0.233 | 0.404 | Pearson |
|  | Right Anterior Inferior | -0.035 | 0.902 | Pearson |
|  | Right Anterior Superior | -0.302 | 0.274 | Pearson |
|  | Right Posterior | -0.365 | 0.181 | Pearson |
|  | Right Tubular Inferior | -0.501 | 0.057 | Pearson |
|  | Right Tubular Superior | -0.286 | 0.301 | Spearman |
|  | Left Anterior Inferior | 0.064 | 0.821 | Pearson |
|  | Left Anterior Superior | -0.288 | 0.297 | Pearson |
|  | Left Posterior | -0.027 | 0.923 | Pearson |
|  | Left Tubular Inferior | -0.228 | 0.415 | Pearson |
|  | Left Tubular Superior | -0.574 | 0.056 | Pearson |
| STOP-BANG | Whole Hypothalamus | 0.541 | 0.060 | Pearson |
|  | Whole Right Hypothalamus | 0.571 | 0.057 | Pearson |
|  | Whole Left Hypothalamus | 0.512 | 0.052 | Pearson |
|  | Right Anterior Inferior | 0.497 | 0.100 | Pearson |
|  | Right Anterior Superior | 0.457 | 0.135 | Pearson |
|  | Right Posterior | 0.540 | 0.070 | Pearson |
|  | Right Tubular Inferior | 0.572 | 0.057 | Pearson |
|  | Right Tubular Superior | 0.494 | 0.061 | Spearman |
|  | Left Anterior Inferior | 0.002 | 0.994 | Pearson |
|  | Left Anterior Superior | 0.198 | 0.538 | Pearson |
|  | Left Posterior | 0.561 | 0.058 | Pearson |
|  | Left Tubular Inferior | 0.574 | 0.056 | Pearson |
|  | Left Tubular Superior | 0.340 | 0.279 | Pearson |
| ESS | Whole Hypothalamus | 0.271 | 0.330 | Pearson |
|  | Whole Right Hypothalamus | -0.194 | 0.491 | Pearson |
|  | Whole Left Hypothalamus | 0.319 | 0.266 | Pearson |
|  | Right Anterior Inferior | 0.501 | 0.057 | Pearson |
|  | Right Anterior Superior | 0.224 | 0.423 | Pearson |
|  | Right Posterior | -0.161 | 0.325 | Pearson |
|  | Right Tubular Inferior | -0.054 | 0.843 | Pearson |
|  | Right Tubular Superior | 0.096 | 0.735 | Spearman |
|  | Left Anterior Inferior | -0.172 | 0.543 | Pearson |
|  | Left Anterior Superior | 0.401 | 0.133 | Pearson |
|  | Left Posterior | -0.073 | 0.795 | Pearson |
|  | Left Tubular Inferior | -0.228 | 0.415 | Pearson |
|  | Left Tubular Superior | 0.347 | 0.205 | Pearson |
| *p<0.05 was considered significant | | | | |

| Supplementary Table 3. Correlations between PSG characteristics and hypothalamic volumes in OSA | | | | |
| --- | --- | --- | --- | --- |
| Correlation Between | | **Correlation Coefficient (R)** | **p-value^*^** | **Correlation Method** |
| AHI | Whole Hypothalamus | 0.021 | 0.940 | Spearman |
|  | Whole Right Hypothalamus | 0.364 | 0.182 | Spearman |
|  | Whole Left Hypothalamus | -0.232 | 0.405 | Spearman |
|  | Right Anterior Inferior | 0.139 | 0.621 | Spearman |
|  | Right Anterior Superior | 0.286 | 0.302 | Spearman |
|  | Right Posterior | 0.439 | 0.101 | Spearman |
|  | Right Tubular Inferior | -0.089 | 0.752 | Spearman |
|  | Right Tubular Superior | -0.104 | 0.713 | Spearman |
|  | Left Anterior Inferior | 0.461 | 0.084 | Spearman |
|  | Left Anterior Superior | -0.236 | 0.398 | Spearman |
|  | Left Posterior | 0.121 | 0.666 | Spearman |
|  | Left Tubular Inferior | -0.393 | 0.147 | Spearman |
|  | Left Tubular Superior | 0.050 | 0.860 | Spearman |
| ODI | Whole Hypothalamus | -0.043 | 0.879 | Spearman |
|  | Whole Right Hypothalamus | 0.310 | 0.261 | Pearson |
|  | Whole Left Hypothalamus | -0.282 | 0.308 | Spearman |
|  | Right Anterior Inferior | -0.018 | 0.950 | Pearson |
|  | Right Anterior Superior | 0.064 | 0.820 | Spearman |
|  | Right Posterior | 0.421 | 0.119 | Pearson |
|  | Right Tubular Inferior | -0.154 | 0.585 | Spearman |
|  | Right Tubular Superior | 0.038 | 0.892 | Pearson |
|  | Left Anterior Inferior | 0.425 | 0.114 | Pearson |
|  | Left Anterior Superior | -0.338 | 0.217 | Pearson |
|  | Left Posterior | 0.177 | 0.528 | Pearson |
|  | Left Tubular Inferior | -0.218 | 0.436 | Pearson |
|  | Left Tubular Superior | -0.037 | 0.897 | Pearson |
| SI | Whole Hypothalamus | -0.029 | 0.919 | Spearman |
|  | Whole Right Hypothalamus | -0.179 | 0.522 | Pearson |
|  | Whole Left Hypothalamus | 0.000 | 1 | Spearman |
|  | Right Anterior Inferior | 0.097 | 0.732 | Pearson |
|  | Right Anterior Superior | -0.286 | 0.302 | Spearman |
|  | Right Posterior | 0.111 | 0.694 | Pearson |
|  | Right Tubular Inferior | -0.339 | 0.216 | Spearman |
|  | Right Tubular Superior | -0.263 | 0.344 | Pearson |
|  | Left Anterior Inferior | 0.047 | 0.867 | Pearson |
|  | Left Anterior Superior | 0.096 | 0.735 | Pearson |
|  | Left Posterior | -0.156 | 0.579 | Pearson |
|  | Left Tubular Inferior | -0.247 | 0.375 | Pearson |
|  | Left Tubular Superior | -0.078 | 0.782 | Pearson |
| AI | Whole Hypothalamus | -0.009 | 0.975 | Spearman |
|  | Whole Right Hypothalamus | 0.113 | 0.689 | Spearman |
|  | Whole Left Hypothalamus | -0.073 | 0.795 | Spearman |
|  | Right Anterior Inferior | -0.240 | 0.390 | Spearman |
|  | Right Anterior Superior | 0.018 | 0.950 | Spearman |
|  | Right Posterior | 0.186 | 0.507 | Spearman |
|  | Right Tubular Inferior | -0.109 | 0.699 | Spearman |
|  | Right Tubular Superior | -0.288 | 0.298 | Spearman |
|  | Left Anterior Inferior | 0.061 | 0.830 | Spearman |
|  | Left Anterior Superior | -0.236 | 0.397 | Spearman |
|  | Left Posterior | 0.193 | 0.490 | Spearman |
|  | Left Tubular Inferior | -0.163 | 0.562 | Spearman |
|  | Left Tubular Superior | -0.066 | 0.815 | Spearman |
| REM Latency | Whole Hypothalamus | -0.066 | 0.831 | Spearman |
|  | Whole Right Hypothalamus | 0.158 | 0.606 | Pearson |
|  | Whole Left Hypothalamus | -0.099 | 0.748 | Spearman |
|  | Right Anterior Inferior | -0.549 | 0.052 | Pearson |
|  | Right Anterior Superior | -0.060 | 0.845 | Spearman |
|  | Right Posterior | 0.314 | 0.296 | Pearson |
|  | Right Tubular Inferior | 0.071 | 0.817 | Spearman |
|  | Right Tubular Superior | 0.010 | 0.973 | Pearson |
|  | Left Anterior Inferior | -0.043 | 0.890 | Pearson |
|  | Left Anterior Superior | -0.164 | 0.592 | Pearson |
|  | Left Posterior | 0.259 | 0.393 | Pearson |
|  | Left Tubular Inferior | 0.249 | 0.411 | Pearson |
|  | Left Tubular Superior | 0.067 | 0.829 | Pearson |
| Minimum SpO2 | Whole Hypothalamus | 0.338 | 0.217 | Spearman |
|  | Whole Right Hypothalamus | 0.000 | 0.999 | Pearson |
|  | Whole Left Hypothalamus | 0.551 | 0.033 | Spearman |
|  | Right Anterior Inferior | -0.274 | 0.323 | Pearson |
|  | Right Anterior Superior | 0.063 | 0.824 | Spearman |
|  | Right Posterior | 0.066 | 0.815 | Pearson |
|  | Right Tubular Inferior | 0.220 | 0.430 | Spearman |
|  | Right Tubular Superior | 0.168 | 0.550 | Pearson |
|  | Left Anterior Inferior | -0.207 | 0.459 | Pearson |
|  | Left Anterior Superior | 0.227 | 0.415 | Pearson |
|  | Left Posterior | 0.201 | 0.473 | Pearson |
|  | Left Tubular Inferior | 0.596 | 0.019 | Pearson |
|  | Left Tubular Superior | 0.151 | 0.591 |  |
| Mean Spo2 | Whole Hypothalamus | -0.213 | 0.447 | Spearman |
|  | Whole Right Hypothalamus | -0.510 | 0.052 | Spearman |
|  | Whole Left Hypothalamus | 0.081 | 0.773 | Spearman |
|  | Right Anterior Inferior | -0.076 | 0.788 | Spearman |
|  | Right Anterior Superior | 0.224 | 0.423 | Spearman |
|  | Right Posterior | -0.412 | 0.127 | Spearman |
|  | Right Tubular Inferior | -0.329 | 0.231 | Spearman |
|  | Right Tubular Superior | -0.022 | 0.937 | Spearman |
|  | Left Anterior Inferior | -0.213 | 0.447 | Spearman |
|  | Left Anterior Superior | 0.294 | 0.287 | Spearman |
|  | Left Posterior | 0.154 | 0.585 | Spearman |
|  | Left Tubular Inferior | 0.013 | 0.963 | Spearman |
|  | Left Tubular Superior | -0.287 | 0.300 | Spearman |
| N1 | Whole Hypothalamus | -0.068 | 0.810 | Spearman |
|  | Whole Right Hypothalamus | -0.017 | 0.592 | Pearson |
|  | Whole Left Hypothalamus | -0.075 | 0.791 | Spearman |
|  | Right Anterior Inferior | -0.112 | 0.690 | Pearson |
|  | Right Anterior Superior | 0.032 | 0.909 | Spearman |
|  | Right Posterior | 0.043 | 0.880 | Pearson |
|  | Right Tubular Inferior | 0.064 | 0.820 | Spearman |
|  | Right Tubular Superior | -0.278 | 0.317 | Pearson |
|  | Left Anterior Inferior | -0.239 | 0.391 | Pearson |
|  | Left Anterior Superior | -0.139 | 0.620 | Pearson |
|  | Left Posterior | 0.117 | 0.679 | Pearson |
|  | Left Tubular Inferior | -0.259 | 0.352 | Pearson |
|  | Left Tubular Superior | 0.120 | 0.670 | Pearson |
| N2 | Whole Hypothalamus | 0.021 | 0.940 | Spearman |
|  | Whole Right Hypothalamus | -0.067 | 0.813 | Pearson |
|  | Whole Left Hypothalamus | 0.021 | 0.940 | Spearman |
|  | Right Anterior Inferior | 0.087 | 0.759 | Pearson |
|  | Right Anterior Superior | -0.146 | 0.603 | Spearman |
|  | Right Posterior | -0.088 | 0.754 | Pearson |
|  | Right Tubular Inferior | 0.046 | 0.869 | Spearman |
|  | Right Tubular Superior | 0.165 | 0.557 | Pearson |
|  | Left Anterior Inferior | 0.130 | 0.643 | Pearson |
|  | Left Anterior Superior | 0.029 | 0.919 | Pearson |
|  | Left Posterior | -0.249 | 0.372 | Pearson |
|  | Left Tubular Inferior | 0.150 | 0.593 | Pearson |
|  | Left Tubular Superior | 0.053 | 0.851 | Pearson |
| N3 | Whole Hypothalamus | 0.092 | 0.745 | Spearman |
|  | Whole Right Hypothalamus | -0.102 | 0.719 | Pearson |
|  | Whole Left Hypothalamus | 0.254 | 0.361 | Spearman |
|  | Right Anterior Inferior | -0.045 | 0.873 | Pearson |
|  | Right Anterior Superior | -0.162 | 0.325 | Spearman |
|  | Right Posterior | -0.221 | 0.429 | Pearson |
|  | Right Tubular Inferior | 0.094 | 0.740 | Spearman |
|  | Right Tubular Superior | 0.111 | 0.695 | Pearson |
|  | Left Anterior Inferior | -0.056 | 0.844 | Pearson |
|  | Left Anterior Superior | 0.283 | 0.308 | Pearson |
|  | Left Posterior | 0.071 | 0.800 | Pearson |
|  | Left Tubular Inferior | 0.386 | 0.155 | Pearson |
|  | Left Tubular Superior | -0.273 | 0.325 | Pearson |
| REM | Whole Hypothalamus | 0.375 | 0.168 | Spearman |
|  | Whole Right Hypothalamus | 0.412 | 0.127 | Pearson |
|  | Whole Left Hypothalamus | 0.107 | 0.704 | Spearman |
|  | Right Anterior Inferior | 0.220 | 0.432 | Pearson |
|  | Right Anterior Superior | 0.325 | 0.237 | Spearman |
|  | Right Posterior | 0.415 | 0.124 | Pearson |
|  | Right Tubular Inferior | -0.168 | 0.550 | Spearman |
|  | Right Tubular Superior | 0.395 | 0.145 | Pearson |
|  | Left Anterior Inferior | 0.584 | 0.022 | Pearson |
|  | Left Anterior Superior | 0.049 | 0.863 | Pearson |
|  | Left Posterior | 0.222 | 0.426 | Pearson |
|  | Left Tubular Inferior | -0.016 | 0.954 | Pearson |
|  | Left Tubular Superior | -0.242 | 0.385 | Pearson |
| *p<0.05 was considered significant | | | | |
